# Method of successive dichotomizations: An improved method for estimating measures of latent variables from rating scale data

**DOI:** 10.1371/journal.pone.0206106

**Published:** 2018-10-18

**Authors:** Chris Bradley, Robert W. Massof

**Affiliations:** Department of Ophthalmology, Johns Hopkins University School of Medicine, Baltimore, Maryland, United States of America; Centre Hospitalier Regional Universitaire de Tours, FRANCE

## Abstract

The most commonly used models for estimating measures of latent variables from polytomous rating scale data are the Andrich rating scale model and the Samejima graded response model. The Andrich model has the undesirable property of estimating disordered rating category thresholds, and users of the model are advised to manipulate data to force thresholds to come out ordered. The Samejima model estimates ordered thresholds, but has the undesirable property of estimating person measures on a non-invariant scale—the scale depends on which items a person rates and makes comparisons across people difficult. We derive the rating scale model logically implied by the generally agreed upon definition of rating scale—a real line partitioned by ordered thresholds into ordered intervals called rating categories—and show that it estimates ordered thresholds as well as person and item measures on an invariant scale. The derived model turns out to be a special case of the Samejima model, but with no item discrimination parameter and with common thresholds across items. All parameters in our model are estimated using a fast and efficient method called the Method of Successive Dichotomizations, which applies the dichotomous Rasch model as many times as there are thresholds and demonstrates that the derived model is a polytomous Rasch model that estimates ordered thresholds. We tested both the Method of Successive Dichotomizations and the Andrich model against simulated rating scale data and found that the estimated parameters of our model were nearly perfectly correlated with the true values, while estimated thresholds of the Andrich model became negatively correlated with the true values as the number of rating categories increased. Our method also estimates parameters on a scale that remains invariant to the number of rating categories, in contrast to the Andrich model.

## Introduction

Measures of latent variables are often estimated from rating scale data. For example, in medical research it is common for disease severity, level of disability or health related quality of life to be estimated on an interval scale from a set of ordinal ratings that patients assign to items on health status questionnaires [1]. Examples include patient reported outcome measures (PROM) such as the SF-36 [2], and clinician judgments of patient signs, symptoms or functional ability such as the Functional Independence Measure [3], Hamilton Depression Rating Scale [4] and Global Assessment of Functioning [5]. The magnitude of a latent disease severity variable may also be estimated from combinations of ordinal ratings and continuous measurements of physical variables, with each observation being treated as a different item [6].

Ordinal ratings for all person-item combinations are analyzed using a probabilistic conjoint measurement model to estimate the magnitude of the latent trait for each person (called the *person measure*) and the sensitivity to that trait of each item (called the *item measure*). The two models most commonly used to estimate person and item measures from ordinal ratings are the Andrich rating scale model [7] (a polytomous Rasch model) and the Samejima graded response model [8] (a polytomous Item Response Theory [IRT] model). Both models also estimate rating category “thresholds”, which are the estimated boundaries between neighboring rating categories. Because rating categories are by definition ordered, rating category thresholds should also be ordered. The Samejima model always estimates ordered thresholds while the Andrich model often estimates disordered thresholds. For example, the Andrich model might estimate the boundary between rating categories 2 and 3 to lie below rather than above the estimated boundary between rating categories 1 and 2.

Over the past three decades the Andrich model has been repeatedly challenged because of its disordered thresholds problem [9–12]. Proponents of the Andrich model interpret model estimates of disordered thresholds as prima fascia evidence of problems with the data [13,14]. They speculate that the putative data problems are a consequence of a flawed rating scale design that gives respondents more categories than they can discriminate reliably. This line of reasoning is circular: thresholds are expected to be ordered, so when the model generates disordered thresholds it must mean there is a problem with the data. What is not being considered in this reasoning is that disordered thresholds are actually pointing to a problem with the model. The problem is not just an academic one. When estimated thresholds come out disordered, users are advised to merge two neighboring rating categories of their choice into a single rating category (equivalent to removing a threshold) and redo the analysis as many times as necessary until all remaining threshold estimates come out ordered. This post hoc process of rescoring data for the explicit purpose of estimating ordered thresholds is simply data manipulation to fit expectations. Because of the popularity of the Andrich model, such post hoc rescoring of data is being practiced across a wide range of fields. For example, in health care research, rescoring has been applied to the development and validation of questionnaires for Parkinsons disease, lung cancer, rheumatoid arthritis and weight loss, as well as many other disorders [15–23].

Technically, both the Samejima model and the Andrich model estimate average thresholds and not the thresholds used on each trial. However, one can prove from the mathematical definition of rating scale—a real line partitioned by ordered thresholds into ordered intervals called rating categories—that average thresholds must be ordered if thresholds on every trial are ordered. To prove this, suppose that on trial k a person rates an item using a rating scale defined by L > 0 individual thresholds {τ_h,k_: 1 ≤ h ≤ L} where τ_h,k_ < τ_h+1,k_ for all h. These L ordered thresholds partition the real line into L + 1 rating categories: C_0_ = (−∞, τ_1,k_), C_h_ = [τ_h,k_, τ_h+1,k_) for 1 ≤ h ≤ L − 1, and C_L_ = [τ_h,k_, ∞), where we use half-open intervals to ensure that every point on the real line belongs to precisely one rating category. Because τ_h,k_ < τ_h+1,k_ for all h on every trial k, average rating category thresholds for *N* trials must satisfy $\tau_{\text{h}}=\frac{1}{\text{N}}\sum_{\text{k}=1}^{\text{N}}\tau_{\text{h},\text{k}}<\frac{1}{\text{N}}\sum_{\text{k}=1}^{\text{N}}\tau_{\text{h}+1,\text{k}}=\tau_{\text{h}+1}$ for all h. Thus, no rating scale model should ever estimate disordered thresholds.

The primary alternative to the Andrich model and other polytomous Rasch models is the Samejima model, an IRT model that always estimates ordered thresholds. The primary objection to the Samejima model is that, unlike the Andrich model, its estimated person and item measures lie on a scale where the unit of measurement can change depending on the item, which makes the interpretation of the estimated scale problematic. Each item lies on its own scale as a consequence of the model’s item discrimination parameter which converts what would otherwise be a measurement model into a descriptive statistical model that violates a fundamental principle of measurement called noninteractive conjoint additivity [24, 25]. Valid Rasch models, including the Andrich model, are constrained to estimate all person and item measures on an invariant scale.

Another problem with the Samejima model is that it combines all sources of trial by trial deviations from its estimated person measures, item measures and thresholds into a single error term without explicitly showing what constraints are placed on the individual sources of error. For example, it is not clear what assumptions the Samejima model makes about the locations of individual thresholds on any trial. Thus, while the Samejima model can be easily modified to estimate scale-invariant measures by removing its item discrimination parameter, a derivation of the model from plausible and generally agreed upon assumptions about the sources and nature of variability in observed responses to ordinal rating scale instruments has yet to be presented.

The primary goal of this paper is to derive a rating scale model that satisfies the basic principles of measurement from two basic assumptions: 1) the generally agreed upon mathematical definition of rating scale, and 2) an assumption about how deviates from estimated person measures, item measures and thresholds are distributed as the number of trials increases. The derived model turns out to be a special case of the Samejima model. We show that all parameters in the derived model can be estimated through repeated application of the dichotomous Rasch model, which is the model both the Samejima and Andrich models reduce to when there are only two rating categories. This new estimation method, called the Method of Successive Dichotomization (MSD) shows that the derived model is a polytomous Rasch model that estimates ordered thresholds. We tested MSD against the Andrich model using simulated rating scale data that ensured there was "no problem with the data". Despite there being no problem with the data, the Andrich model still estimated disordered thresholds. All estimated parameters of MSD were nearly perfectly correlated with the true values.

## Methods

### Deriving a rating scale model from first principles

The derivation of any rating scale model should begin with a definition of rating scale. The generally accepted mathematical definition of rating scale is a real line partitioned by ordered thresholds into ordered intervals called rating categories. To ensure that every point on the real line belongs to precisely one rating category, the L + 1 rating categories on trial *k* are defined to be C_0_ = (−∞, τ_1,k_), C_h_ = [τ_h,k_, τ_h+1,k_) for 1 ≤ h ≤ L − 1, and C_L_ = [τ_L,k_, ∞). Thresholds {τ_h,k_: 1 ≤ h ≤ L} may vary from trial to trial, but on every trial they must remain ordered. This assumption of threshold ordering may seem unassuming, but as we will show later the Andrich model does not require thresholds to be ordered on any trial.

The rating person *i* assigns to item *j* on trial *k* depends on the person measure *β*_*i*,*k*_, the item measure *δ*_*j*,*k*_, and the set of thresholds {τ_h,k_: 1 ≤ h ≤ L} used by person *i* on trial *k*. For convenience, we will let subscript *k* in τ_h,k_ be a universal index across all trials and all persons in the sample. Thus, if there are *N* persons and *M* items and each person rates each item just once, then *k* ∈ {1, …, *NM*}. By convention, the rating person *i* assigns to item *j* on trial *k* is the rating category within which *β*_*i*,*k*_ − *δ*_*j*,*k*_ lies (rather than the interval in which *δ*_*j*,*k*_ − *β*_*i*,*k*_ lies). Conceptually, *β*_*i*,*k*_ − *δ*_*j*,*k*_ represents a comparison of the magnitude of a person’s trait relative to that of the item.

Our goal is to estimate person measures, item measures and rating category thresholds on an invariant scale. All parameters we wish to estimate are expected values. If we write $\beta_{i,k}=\beta_{i}+\varepsilon_{\beta_{i},k},\delta_{j,k}=\delta_{j}+\varepsilon_{\delta_{j},k}$ and $\tau_{h,k}=\tau_{h}+\varepsilon_{\tau_{h},k}$, where *β*_*i*_, *δ*_*j*_ and *τ*_*h*_ are expected values and $\varepsilon_{\beta_{i},k},\varepsilon_{\delta_{j},k}$ and $\varepsilon_{\tau_{h},k}$ are deviates, then our goal is to estimate *β*_*i*_, *δ*_*j*_ and *τ*_*h*_ for all *i*, *j* and *h*. To estimate all parameters on an invariant scale, it is necessary that the unit of measurement does not depend on which item is rated, which person rates the item, or which rating is assigned to the person/item encounter (*i*.*e*., which thresholds were used on a particular trial). In both the Andrich and Samejima models, the units of measurement are functions of the variance in the distributions of the error terms. Thus, to estimate parameters on an invariant scale, we will assume that as *k* → ∞, the distribution of $\varepsilon_{\beta_{i},k}$ is identical for all persons *i*, the distribution of $\varepsilon_{\delta_{j},k}$ is identical for all items *j*, and the distribution of $\varepsilon_{\tau_{h},k}$ is identical for all thresholds *h*.

With these assumptions, we can calculate the probability of observing any rating. Person *i* will assign the lowest rating *C*_0_ to item *j* when *β*_*i*,*k*_ − *δ*_*j*,*k*_ < *τ*_1,*k*_, or equivalently, when $\beta_{i}-\delta_{j}-\tau_{1}<\varepsilon_{\tau_{1},k}-\varepsilon_{\beta_{i},k}+\varepsilon_{\delta_{j},k}$. Using the shorthand notation *γ*_*ij*_ = *β*_*i*_ − *δ*_*j*_ and defining *φ*(*ε*) to be the distribution of the combined deviate $\varepsilon_{\tau_{h},k}-\varepsilon_{\beta_{i},k}+\varepsilon_{\delta_{j},k}$ as *k* → ∞, the probability *p*_*ij*_(*C*_0_) of person *i* assigning rating *C*_0_ to item *j* becomes

$$p_{ij}\left(C_{0}\right)=\int_{\gamma_{ij}-\tau_{1}}^{\infty }\varphi \left(\varepsilon \right)d\varepsilon$$ (1)

More generally, *φ*(*ε*) represents the distribution of the combined deviate $\varepsilon_{\tau_{h},k}-\varepsilon_{\beta_{i},k}+\varepsilon_{\delta_{j},k}$ for any *h* as *k* → ∞ because the distribution of $\varepsilon_{\tau_{h},k}$ was assumed to be identical for all *h*. This means that $\int_{\gamma_{ij}-\tau_{h}}^{\infty }\varphi (\varepsilon )d\varepsilon$ represents the probability person *i* rates item *j* with some rating *C*_*q*_ where *q* < *h*. Therefore, the difference

$$p_{ij}\left(C_{h}\right)=\int_{\gamma_{ij}-\tau_{h+1}}^{\infty }\varphi \left(\varepsilon \right)d\varepsilon -\int_{\gamma_{ij}-\tau_{h}}^{\infty }\varphi \left(\varepsilon \right)d\varepsilon$$ (2)

specifies the probability of person *i* assigning rating *C*_*h*_ to item *j*, for 1 ≤ *h* ≤ *L* − 1. For the special case where *h* = *L*, we have

$$p_{ij}\left(C_{L}\right)=1-\int_{\gamma_{ij}-\tau_{L}}^{\infty }\varphi \left(\varepsilon \right)d\varepsilon$$ (3)

Eqs 1–3 represent the rating scale model implied by the mathematical definition of rating scale and the assumption that the combined error term has distribution *φ*(*ε*) as *k* → ∞ regardless of which item is rated, which person rated the item, or which rating category was observed. Estimated thresholds are always ordered using Eqs 1–3 because disordered thresholds produce negative probabilities, which are undefined. Thus, all valid sets of candidate thresholds in a maximum likelihood estimation (MLE) using Eqs 1–3 must be ordered.

#### Comparison to the Samejima model

Eqs 1–3 represent a special case of the Samejima model [8] with no item discrimination parameter and with common thresholds across items. Stated differently, Eqs 1–3 are the same as Muraki’s modified graded response model (MGRM) [26] with no item discrimination parameter. The lack of an item discrimination parameter allows the derived model to satisfy a key property of measurement, namely that the unit of measurement remain invariant to the specific set of items each person responded to. Our derivation also demonstrates that the MGRM, and by extension the Samejima model, is consistent with allowing individual thresholds to lie anywhere on each trial as long as they remain ordered. Previously it had been thought that these IRT models imply all thresholds must be perfectly correlated across people [27].

#### Comparison to the Andrich model

The Andrich model [7] makes the same assumptions we made except that it does not require thresholds to be ordered on any trial [28]. When person *i* assigns rating *C*_*h*_ to item *j* on trial *k*, the Andrich model assumes that *β*_*i*,*k*_ − *δ*_*j*,*k*_ lies both above the set of thresholds {*τ*_*q*,*k*_: *q* ≤ *h*} and below the set of thresholds {*τ*_*q*,*k*_: *q* > *h*}. Thus, the Andrich model requires thresholds to be segregated into two different sets, but not ordered. To estimate expected values, the Andrich model assumes that the cumulative logistic function specifies the probability *γ*_*ij*_ = *β*_*i*_ − *δ*_*j*_ lies above or below *τ*_*q*_. The probability *γ*_*ij*_ lies above *τ*_*q*_ becomes

$$p\left(\gamma_{ij}>\tau_{q}\right)=\frac{\text{exp}\left(\gamma_{ij}-\tau_{q}\right)}{1+\text{exp}\left(\gamma_{ij}-\tau_{q}\right)}$$ (4)

and the probability *γ*_*ij*_ lies below *τ*_*q*_, or 1 − *p*(*γ*_*ij*_ > *τ*_*q*_), becomes

$$p\left(\gamma_{ij}<\tau_{q}\right)=\frac{1}{1+\text{exp}\left(\gamma_{ij}-\tau_{q}\right)}$$ (5)

The Andrich model is derived by calculating the probability *p*(*C*_*h*_) that *γ*_*ij*_ lies above all thresholds {*τ*_*q*_: *q* ≤ *h*} and below all thresholds {*τ*_*q*_: *q* > *h*}, assuming all events are statistically independent:

$$p\left(C_{h}\right)=\frac{\prod_{q=1}^{h}p\left(\gamma_{ij}>\tau_{q}\right)\prod_{q=h+1}^{L}p\left(\gamma_{ij}<\tau_{q}\right)}{\sum_{h=0}^{L}\left[\prod_{q=1}^{h}p\left(\gamma_{ij}>\tau_{q}\right)\prod_{q=h+1}^{L}p\left(\gamma_{ij}<\tau_{q}\right)\right]}$$ (6)

where for notational simplicity we define *p*(*γ*_*ij*_ > *τ*_0_) ≡ 1 because *τ*_0_ is undefined. Eq 6 reduces to the more familiar Andrich model equation when Eqs 4 and 5 are substituted in for the corresponding response probabilities:

$$p\left(C_{h}\right)=\frac{\text{exp}\sum_{q=1}^{h}\left(\gamma_{ij}-\tau_{q}\right)}{\sum_{h=0}^{L}\left[\text{exp}\sum_{q=1}^{h}\left(\gamma_{ij}-\tau_{q}\right)\right]}$$ (7)

Because the Andrich model does not require thresholds to be ordered on any trial, it is no surprise that its estimates of expected rating category thresholds frequently come out disordered. We note that Andrich claims that all thresholds of any rating scale model must be ordered on every trial in order to satisfy the requirements of a Guttman scale [29]. However, his model does not satisfy this requirement.

### The method of successive dichotomizations

All parameters in Eqs 1–3 can be estimated in a fast and efficient way through repeated application of the dichotomous Rasch model. We call this method of estimation the Method of Successive Dichotomizations (MSD). MSD is possible because threshold *τ*_*h*_ in Eqs 1–3 is defined to be at $\sum_{q=0}^{h-1}p_{ij}(C_{q})=\sum_{q=h}^{L}p_{ij}(C_{q})=0.5$. This means that merging *C*_*q*−1_ and *C*_*q*_ by removing threshold *τ*_*q*_ ≠ *τ*_*h*_ has no effect on the estimated location of *τ*_*h*_ (up to a choice of axis origin) because *p*_*ij*_(*C*_*q*−1_) ∪ *p*_*ij*_(*C*_*q*_) = *p*_*ij*_(*C*_*q*−1_) + *p*_*ij*_(*C*_*q*_). In other words, as long as we have some means of keeping the axis origin constant, any thresholds that remain after rescoring as well as all other parameters in Eqs 1–3 are invariant to how data are rescored. In particular, we could repeat this rescoring operation *L* − 1 times (given *L* thresholds) until the original data are dichotomized and only a single threshold remains without altering the estimated location of that threshold. In contrast, rescoring with the Andrich model leads to different parameter estimates because *τ*_*h*_ is defined to be at *p*_*ij*_(*C*_*h*−1_) = *p*_*ij*_(*C*_*h*_). If we rescore by merging *C*_*h*_ and *C*_*h*+1_, then *τ*_*h*_ will move to a different location, namely where *p*_*ij*_(*C*_*h*−1_) = *p*_*ij*_(*C*_*h*_) ∪ *p*_*ij*_(*C*_*h*+1_) would have been before rescoring.

Because Eqs 1–3 permit rescoring of data, MSD begins by constructing *L* different dichotomized matrices {*D*_*h*_: 1 ≤ h ≤ L} from the original rating scale data where *D*_*h*_ maps the observed response *r*_*ij*_ of person *i* to item *j* in the original rating scale to 0 if *r*_*ij*_ < *h* and to 1 if *r*_*ij*_ ≥ *h*. For each *D*_*h*_, the dichotomous Rasch model can be used to estimate a person measure ${\hat{\beta }}_{i}(\tau_{h})$ for each person *i* and an item measure ${\hat{\delta }}_{j}(\tau_{h})$ for each item *j*. Final MSD-estimated person and item measures ${\hat{\beta }}_{i}$ and ${\hat{\delta }}_{j}$ are simply the averages of the *L* different estimated person and item measures: ${\hat{\beta }}_{i}=\frac{\sum_{h=1}^{L}{\hat{\beta }}_{i}(\tau_{h})}{L}$ and ${\hat{\delta }}_{j}=\frac{\sum_{h=1}^{L}{\hat{\delta }}_{j}(\tau_{h})}{L}$, with the axis origin chosen by convention to be the mean item measure.

The second step of MSD is to estimate all thresholds. Each threshold can be estimated independently of other thresholds because *D*_*h*_ is known and ${\hat{\beta }}_{i}$ and ${\hat{\delta }}_{j}$ have already been estimated for all *i* and *j*. Specifically, to estimate a given threhold *τ*_*h*_, use Eqs 1–3 with *D*_*h*_ as the rating scale data and with ${\hat{\beta }}_{i}$ and ${\hat{\delta }}_{j}$ as known parameters rather than parameters to be estimated. The only unknown is threshold *τ*_*h*_, which even with a brute force MLE takes little time to estimate. In summary, MSD is a fast and efficient means of estimating all parameters in Eqs 1–3 (code provided [30]) because *L* separate MLE’s are used to estimate the person measures and item measures (via the dichotomous Rasch model) and *L* separate single-parameter estimation MLE’s are used to estimate the thresholds, which is computationally more efficient than a single MLE estimating all person measures, item measures and thresholds.

Standard errors for each MSD estimated person or item measure can be calculated by taking the standard deviation of the *L* individually estimated person or item measures and dividing it by $\sqrt{L}$, which is the degrees of freedom. Note however that in the case of the person measures, we must first subtract the estimated thresholds: the standard errors are calculated on ${\hat{\beta }}_{i}(\tau_{h})-{\hat{\tau }}_{h}$ rather than ${\hat{\beta }}_{i}(\tau_{h})$ because the threshold (unknown in the dichotomous case) is incorporated into the estimated person measure of the dichotomous Rasch model. Standard errors for the thresholds come from the MLE used to estimate the thresholds: the reciprocal of the square root of the Hessian.

MSD has a theoretical significance in addition to a practical one: it demonstrates that a special case of the Samejima model is a polytomous Rasch model. There is no unique extension of the dichotomous Rasch model because different assumptions can be made about how two or more thresholds are distributed on any trial. Eqs 1–3 represent the extension of the dichtomous Rasch model to the polytomous case when thresholds are required to be ordered on every trial. The Andrich model represents the extension of the dichtomous Rasch model to the polytomous case when thresholds are required to be segregated, but not ordered, on every trial.

### Simulations to test the models

There is currently no known way to directly observe the neural representations of thresholds, rating scales, or the decision making process that leads to the assignment of ratings to items. Thus, the only way to test the "accuracy" of different rating scale models is to compare parameter estimates to their true values in simulations. Every simulation is based on assumptions, and the key assumption in our simulation is that thresholds must be ordered on every trial. This is an assumption that Andrich agrees with even if his model is logically inconsistent with it. We also ensured that the distributions of all threshold deviates were identical across trials, and that simulated persons had no trouble assigning ratings to items because every point on the real line belonged to precisely one rating category. In other words, there was no "problem with the data" as defenders of the Andrich model often claim when the Andrich model estimates disordered thresholds.

The method we used for generating ordered thresholds on every trial requires some explanation because the method commonly used—on each trial, keep randomly selecting a set of thresholds until by chance all thresholds are ordered—leads to threshold distributions that differ from the distributions they were initially drawn from. For example, thresholds randomly drawn from normal distributions will have skewed distributions if one discards all sets of disordered thresholds. Our method ensures that thresholds have the desired threshold distribution across all trials. We emphasize that our method is not in any way meant to represent how humans select thresholds and is only a means of generating sets of thresholds with desired properties.

Let *φ*_*τ*_(*ε*) represent the desired distribution of threshold deviates and let {τ_h_: 1 ≤ h ≤ L} represent the desired means of the threshold distributions. Our goal will be to produce *K* sets of *L* ordered thresholds that are not perfectly correlated with each other such that the distribution of deviates for each threshold approaches *φ*_*τ*_(*ε*) as *K* → ∞. The method has two parts. First, we will create *K* sets of *L* ordered thresholds with the desired threshold distributions but where all thresholds are perfectly correlated with each other. Then, we will decorrelate those thresholds while preserving the desired threshold distributions. Begin by randomly selecting *K* deviates from *φ*_*τ*_(*ε*). For each deviate, add the set of *L* threshold means {τ_h_} to create *K* sets of *L* ordered thresholds that are perfectly correlated with each other. Thresholds can now be decorrelated without altering the threshold distributions by repeating the following process a sufficient number of times (*KL* iterations were sufficient for our simulations): 1) randomly choose two of the *K* sets, say sets *S*_*i*_ and *S*_*j*_ where 1 ≤ *i* < *j* < *K*, then 2) randomly choose an *h* where 1 ≤ h ≤ L, and 3) switch threshold *h* in *S*_*i*_ with threshold *h* in *S*_*j*_ only if switching preserves ordering; otherwise do not modify *S*_*i*_ and *S*_*j*_. This iterative procedure allows us to generate *K* sets of *L* ordered thresholds that are not perfectly correlated with each other such that the deviates for each threshold have the same distribution *φ*_*τ*_(*ε*) as *K* → ∞.

## Results

To test MSD and the Andrich model, we simulated rating scale data in MATLAB (code provided [30]) for *N* = 1000 persons, *M* = 100 items and *L* = 9 thresholds (10 rating categories), with the probability of missing data set to 10%. All true person measures, item measures and thresholds were randomly chosen from a uniform distribution over the interval [−2,2] with the mean item measure set to zero; thresholds were ordered (relabeled) only after being randomly chosen. Two types of error terms were added: a trial-by-trial error term placed on *β*_*i*,*k*_ − *δ*_*j*,*k*_ and a within-person threshold error term that guaranteed thresholds were different from trial to trial. Both error terms were chosen from the standard normal distribution. Between-person threshold error terms representing differences in average thresholds between persons were not added after we confirmed that neither model can distinguish between a between-person threshold error term and a difference in true person measures.

**Fig 1:**
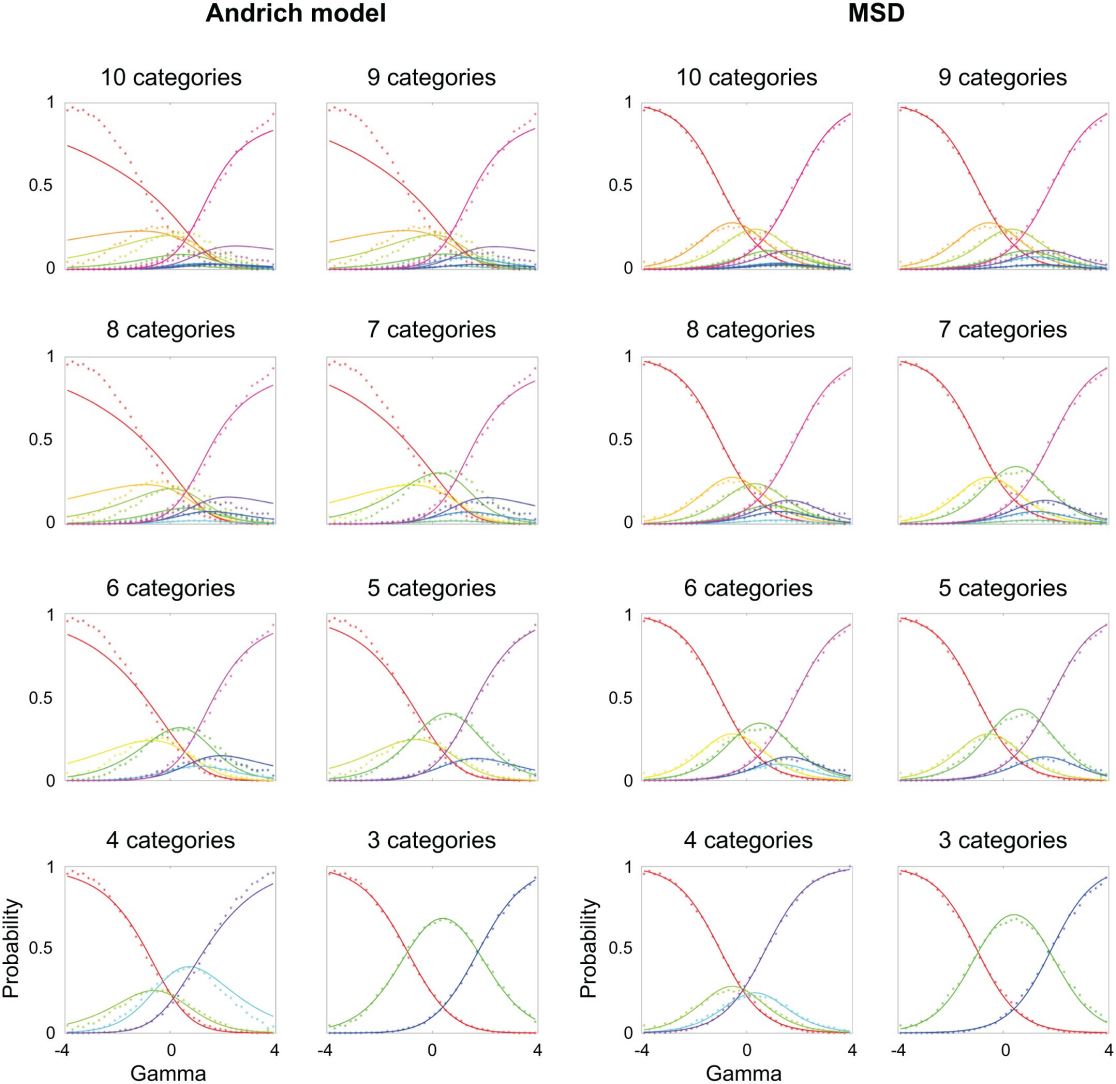
Comparing probability curves for the Andrich model and MSD. Actual frequencies (dots) of observing rating categories as a function of differences in true person and item measures, or gamma, compared to the predicted values (curves) from the Andrich model (left) and MSD (right). Binning of gamma was done for the simulated data in intervals of 0.2 logits. The Andrich model becomes less accurate than MSD the greater the number of rating categories.

We estimated parameters using both MSD (code provided [30]) and Winsteps (software implementing the Andrich model) and compared estimated values to the true values. Thresholds came out disordered for the Andrich model, which led us to rescore data post hoc and reduce the number of thresholds to *L* = 8. Once again, we compared MSD to Winsteps, and continued this rescoring process and comparison process until Andrich model thresholds came out ordered, which finally occurred at *L* = 2 thresholds. Fig 1 shows the probability curves (solid curves)—predicted frequencies for differences between person and item measures—for the Andrich model and MSD, with the true values (dots) also plotted for comparison, for all cases from *L* = 9 to *L* = 2 (at *L* = 1 both models reduce to the same dichotomous Rasch model and there is nothing to compare). The "accuracy" of MSD was much higher than that of the Andrich model when the number of rating categories was large, while both models exhibited similar accuracy with a small number of rating categories.

**Fig 2:**
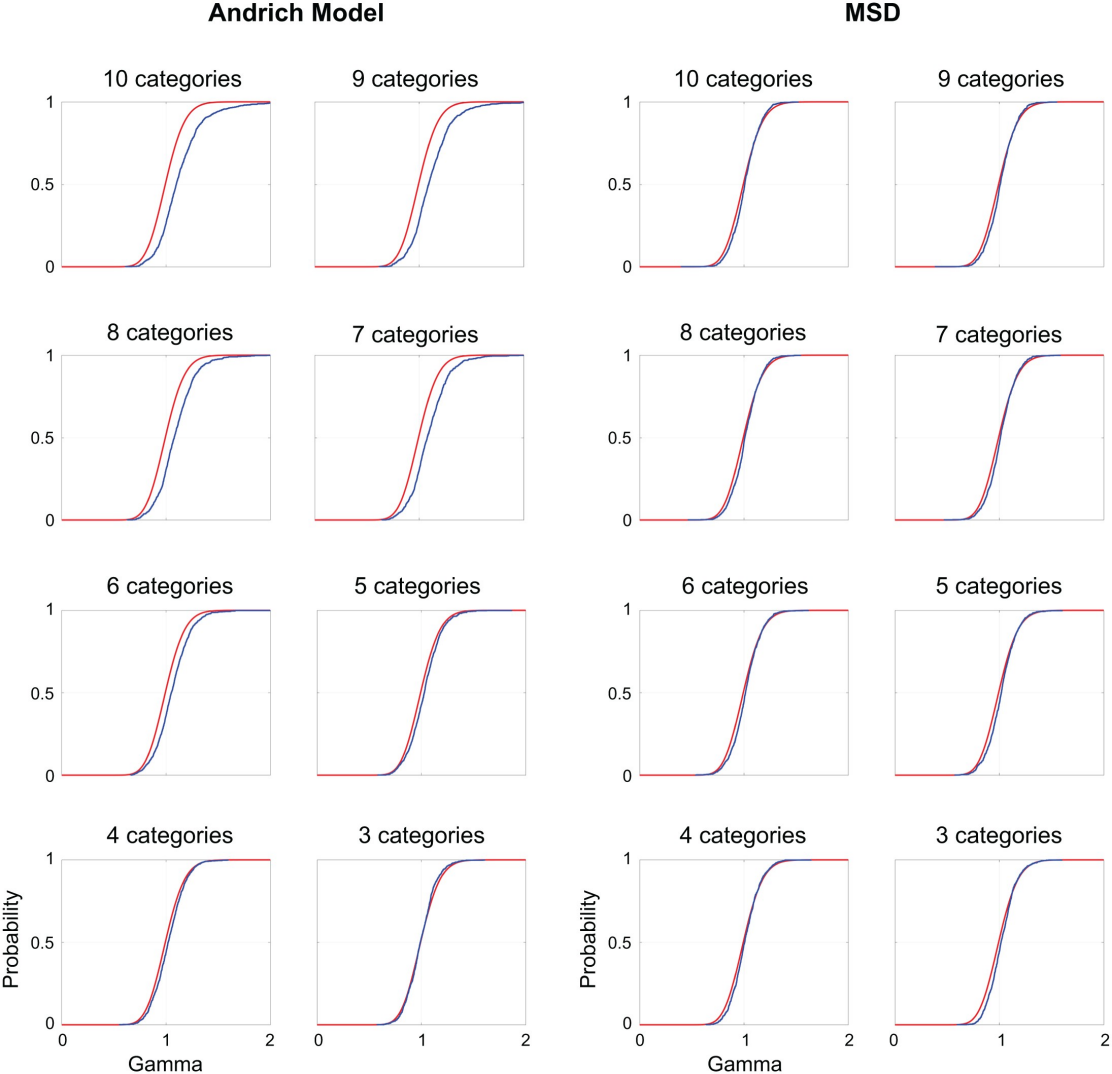
Comparing person infit mean squares for the Andrich model and MSD. Cumulative distribution functions of the person infit mean squares (blue curves) are compared to cumulative distribution functions of chi square divided by degrees of freedom (red curves), for the Andrich model (left) and MSD (right). The red and blue curves should be identical under the assumption that the only source of variance is random error. MSD satisfies this assumption better than the Andrich model as the number of rating categories increases.

Fig 2 compares the cumulative distribution function of the person "infit mean squares" (blue curves)–the ratio of the observed response variance to the expected variance, across items for each person—to the cumulative distribution function of chi squared over degrees of freedom (red curves) for both models from *L* = 9 to *L* = 2. The two cumulative distribution functions should be identical if the assumption of unidimentionality is satisfied and the only source of variance is random error [27]. MSD satisfies this assumption (an a priori assumption in our simulations) better than the Andrich model when the number of rating categories increases.

**Fig 3:**
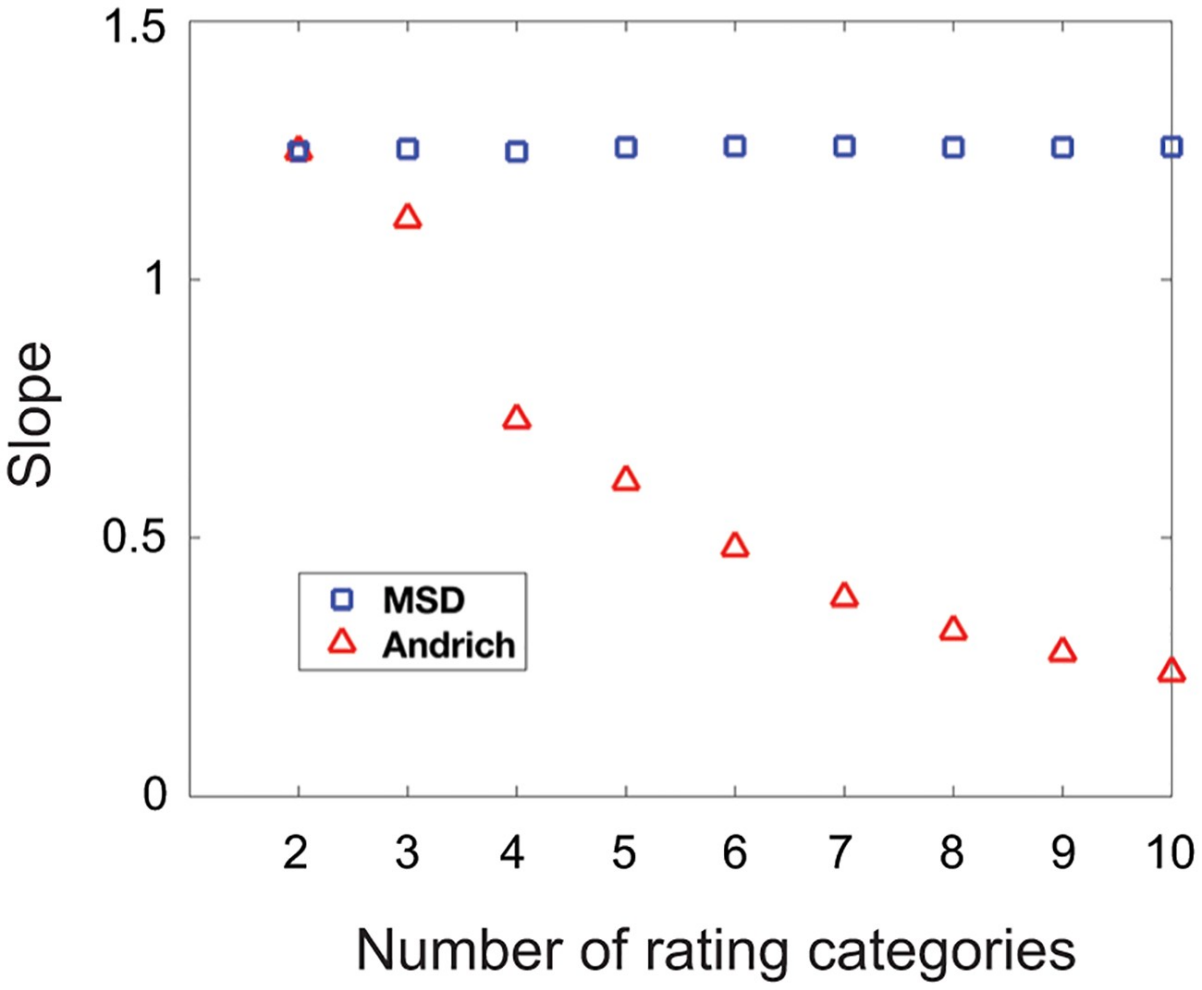
Slopes of the best-fitting line between estimated and true item measures. Slopes of the best-fitting line between estimated and true item measures for MSD (blue squares) and the Andrich model (red triangles) are plotted as a function of the number of rating categories. MSD estimates parameters on the same scale regardless of the number of rating categories because it only uses the dichotomous Rasch model, while the Andrich model estimates parameters on a scale that is dependent on the number of rating categories.

Fig 3 shows a key difference between the two models that has important practical consequences: the slope of the best-fitting line between estimated and true item measures changes as a function of the number of rating categories for the Andrich model while the slope remains invariant for MSD. This means that rescoring data with the Andrich model changes the scale on which it estimates parameters, making it difficult or impossible to compare estimated parameters from different studies or across different conditions (e.g. pre- vs. post-treatment if rescoring was done differently in the two conditions). With MSD, estimated parameters are not dependent on the number of rating categories making such comparisons feasible. We note that the scale for both models is sensitive to the combined variance of the error terms added. For example, changing the standard deviation of the error distribution from 1 to 2 will change the scale for both models. However, the effect of doing so can be predicted for MSD because variance in the dichotomous Rasch model (which uses a logistic function) is defined to be $\sigma^{2}=\frac{\pi^{2}}{3a^{2}}$ where *a* is its discrimination index. The sum of the variances in our error terms was *σ*^2^ = 2, giving us a predicted slope of *a* = 1.2826. The average slope for MSD across all 9 simulations in Fig 2 was 1.2546. The small difference between the predicted and average slopes might be the consequence of using a normal distribution to generate error terms in the simulation rather than using the logistic distribution assumed by the model.

**Fig 4:**
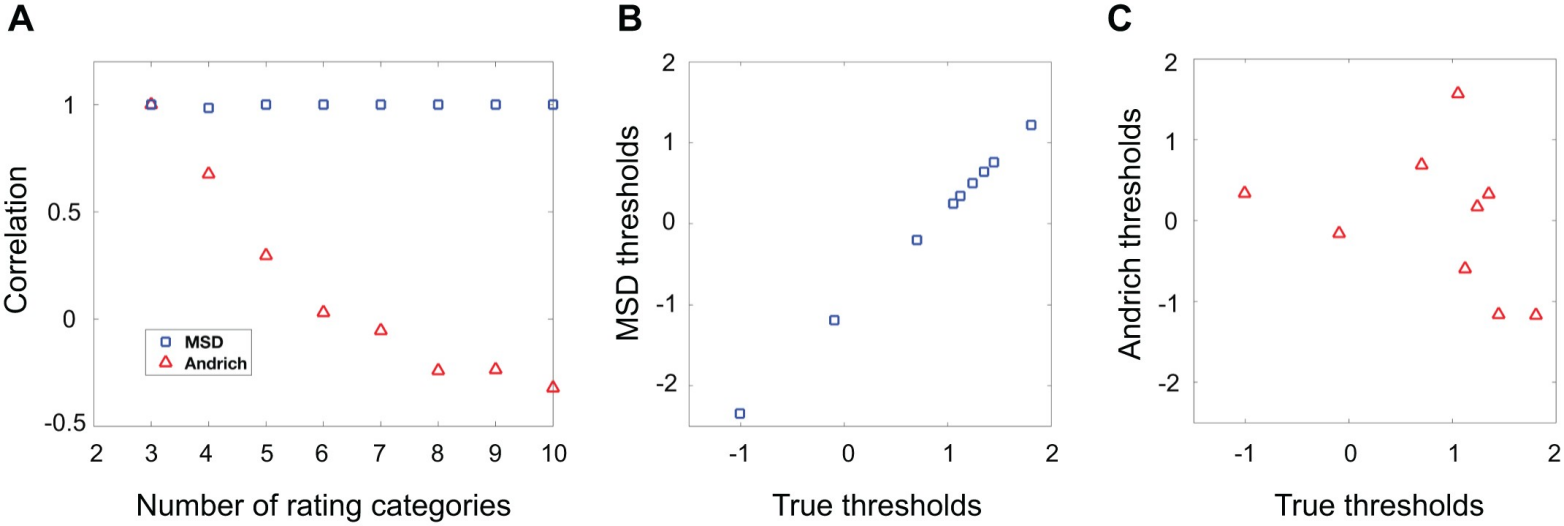
Comparing estimated vs. true thresholds for the Andrich model and MSD. Correlations between estimated thresholds and true thresholds for MSD (blue squares) and the Andrich model (red triangles). Fig 4A shows that the correlation between estimated and true thresholds is nearly perfect for MSD, with *r* > 0.999 across all conditions except when there were 4 rating categories where *r* = 0.985. The correlation between thresholds estimated by the Andrich model and the true thresholds turned negative with a sufficiently high number of rating categories. Fig 4B shows an example of MSD thresholds vs. true thresholds with 10 rating categories, and Fig 4C shows an example of Andrich thresholds vs. true thresholds for the same condition.

Fig 4A shows the correlations between estimated and true thresholds for both models as a function of the number of rating categories. MSD-estimated thresholds were in nearly perfect agreement with the true thresholds regardless of the number of categories (*r*^2^ > 0.998 across all conditions except with 4 rating categories where *r*^2^ = 0.97) while Andrich model thresholds were negatively correlated with the true thresholds when the number of rating categories became large. This negative correlation is not specific to this particular simulation. As the number of thresholds increases, the number of rating categories that are used infrequently increases, and because Andrich model thresholds are defined to be at the crossing points between neighboring probability curves (i.e. where *p*_*ij*_(*C*_*h*−1_) = *p*_*ij*_(*C*_*h*_) for any *h*), the probability of these crossing points being in reversed order increases with lower frequencies of observing that rating. Fig 4B and 4C show how MSD thresholds and Andrich model thresholds compare to true thresholds when there are 10 rating categories.

Estimated person and item measures from both models (not shown) were nearly perfectly correlated with their true values [31] with a range of 0.9939 ≤ *r*^2^ ≤ 0.9970 for Andrich model item measures, a range of 0.9954 ≤ *r*^2^ ≤ 0.9969 for MSD item measures, a range of 0.9607 ≤ *r*^2^ ≤ 0.9773 for Andrich model person measures, and a range of 0.9484 ≤ *r*^2^ ≤ 0.9729 for MSD person measures. Although data analyzed using the Andrich model result in person and item measure estimates that are linearly related to the true values, they cannot be compared across studies without equating scales. This can be demonstrated by calculating *r*^2^ between all 8 sets of estimated person and item measures (i.e., combining estimates across all 8 conditions into a single set of estimates) and their true values. The combined person measure estimates of the Andrich model had an *r*^2^ = 0.7456 with the true values while MSD person measure estimates had an *r*^2^ = 0.9103. For combined item measure estimates, the Andrich model had an *r*^2^ = 0.7790 while MSD had an *r*^2^ = 0.9966.

## Discussion

Defenders of the Andrich model have argued that estimating disordered thresholds suggests there is a problem with the data, and not with the model, because thresholds *should* be ordered. But if we assume that thresholds are ordered on every trial, then all average thresholds must be ordered. Thus, given the assumptions Andrich himself agrees with, there is no theoretical justification for any rating scale model to estimate disordered thresholds. Conversely, we have shown that the Andrich model itself does not require thresholds to be ordered. This calls into question the practice of manipulating data to force the model to estimate ordered thresholds. Our simulations also ensured there was no "problem with the data", demonstrating that the problem lies with the Andrich model and not with the data.

We have derived the rating scale model logically implied from the generally agreed upon definition of a rating scale as a real line partitioned by ordered thresholds into ordered intervals called rating categories. Our derived model is a special case of the Samejima model with no item discrimination parameter and with common thresholds across items. We also introduced a simple, fast and efficient way of estimating all parameters in our model called the Method of Successive Dichotomizations, or MSD. The approach used in MSD—successively dichotomize the original rating scale data and apply the dichotomous Rasch model each time—is similar in spirit to previous approaches that estimated parameters one rating category at a time [32,33]. However, by repeatedly applying the dichtomous Rasch model, MSD shows that a special case of an IRT model is the correct extension of the dichotomous Rasch model to the polytomous case when all thresholds are required to be ordered on every trial.

MSD is an approximation to an MLE done on all parameters simultaneously, and while its estimated parameters were nearly perfectly correlated with the true values in our simulated rating scale data, its estimates are only as good as the estimates of the dichotomous Rasch model on each dichotomization. In particular, the dichotomizations for the lowest and highest thresholds may lead to unreliable estimates of person and item measures if there are too few ratings in the lowest and/or highest rating categories.

Users of the Andrich model need not redo previous analyses with MSD as long as they are only interested in estimated person and item measures and do not plan on comparing parameter estimates across studies. If however the average rating scale used by respondents is relevant in a study, or if parameter estimates from multiple studies are to be compared, then the Andrich model should not be used. Disordered thresholds do not define a rating scale, and merging neighboring rating categories fundamentally alters the locations of any remaining thresholds in the Andrich model as well as the scale upon which all parameters are estimated.
